# Agenda navigation in consultations covering multiple topics. A qualitative case study from general practice

**DOI:** 10.1080/02813432.2021.1958472

**Published:** 2021-08-26

**Authors:** Ann Dorrit Guassora, May-Lill Johansen, Kirsti Malterud

**Affiliations:** a The Research Unit and Section of General Practice, Department of Public Health, University of Copenhagen, Copenhagen, Denmark; b The Research Unit for General Practice, Department of Community Medicine, Faculty of Health Sciences, UiT The Arctic University of Norway, Tromsø, Norway; c Research Unit for General Practice, NORCE Norwegian Research Centre, Bergen, Norway; d Department of Global Public Health and Primary Care, University of Bergen, Norway

**Keywords:** Physician-patient relations, general practice, communication, linguistics, patient-centered care

## Abstract

**Objective:**

To explore how agenda navigation may be accomplished underway in consultations covering multiple topics, we identified and analyzed one GP’s communicative strategies.

**Design, setting, and subjects:**

A qualitative observational case study with linguistic microanalysis of an exemplary consultation between a female patient with diabetes and her male GP. We used speech act theory to identify communicative actions that indicated agenda navigation by the GP in transitions between episodes concerning ten topics.

**Results:**

Microanalysis revealed different aspects of agenda navigation by the GP using speech acts, especially ways of opening or closing an episode. The opening of episodes was characterized by speech acts accepting the patient’s request to discuss a topic, mostly at the beginning of the consultation. Speech acts to inform or to request information from the patient dominated later in the consultation. The GP closed all episodes using speech acts to instruct or appraise the patient, or to make agreements and plans.

**Conclusion and practice implications:**

Skilful agenda navigation is an important tool for consultations covering multiple issues and could be further developed for medical education. The opening and closing of episodes were vital communicative strategies supporting patient-centered communication in a complex consultation while maintaining the focus of the consultation agenda.

**KEY POINTS**
- While traditional consultation models cover one health problem, GP consultations often include many patient issues in each session.
- Linguistic microanalysis of speech acts helped to identify communication strategies in a GP consultation with multiple topics.
- The GP conducted agenda navigation by distinctly opening and closing episodes concerning specific topics.
- Episodes were opened by accepting, informing, and requesting and closed by instruction, appraisal, making agreements, or plans.

## Background

Consultations in general practice often address more than one problem [1–3]. Still, consultation models commonly simplify the complexity of clinical practice by portraying the process as dealing with a single problem [4], and some GPs attempt to allow only one issue per consultation. For GPs to venture with confidence, and in collaboration with patients, into the handling of several health problems, we need a better knowledge of how to deal with these consultations. As the number of patients with multiple chronic conditions increases, such skills will be even more important.

Deciding which issues to address during a consultation is part of the patient-centered method and is usually called *agenda setting* [5,6]. Gobat et al. [7] established consensus on the core domains of agenda-setting in consultations and additionally proposed the concepts of agenda mapping and agenda navigation. *Agenda mapping* is the identification of the potential content areas for discussion before committing to a course of action for the consultation and prioritizing topics to agree on a focus. It guides the consultation by offering a pattern against which to structure the clinical discourse. While previous models of agenda setting also suggest structuring [5,6,8,9], this model explicitly acknowledges the need to be responsive to new topics that may arise during the patient encounter. Openness to new topics is described as *agenda navigation* by Gobat et al. [7] and, drawing on our own clinical experiences, we suggest that it is relevant for more than the management of new topics. In this article, we take agenda navigation as a broader concept that embraces the communicative processes employed by the GP in recognizing and prioritizing concurrent topics and adjusting the agenda as the consultation is underway. In this context, we do not restrict agenda navigation to health problems, and we include other topics raised by the patient or the GP [10].

In the 1980s, studies of talk between patients and doctors prepared the ground for research in medicine, psychology, and social sciences. Analysis of observations from clinical practice included interactional and discourse functions as well as implications for humane care. Some studies looked at efficient interaction and patient satisfaction [4,11,12], while others emphasized patient-centeredness and equality in communication [13,14]. Inspired by critical theory and feminism, issues of power and gender in the interaction were explored [15,16]. An underlying assumption was that the power asymmetry of medical dialogue granted the doctor control at the patient’s expense [17,18]. Methodologically, interruptions and topic control were at that time seen as linguistic markers of power, representing paternalistic and inappropriate behaviors [19].

Four decades on, we want to challenge the understanding of topic control as unequivocal power conduct, without dismissing the power issues at play. We acknowledge the GP’s responsibility for sensible time management with the potential to accommodate multiple problems and patient-centeredness. Agenda setting offers a conceptual framework for organizing consultations, but the practical skills a GP needs to handle the agenda during interaction in the consultation are less specified. The purpose of the study is to explore how agenda navigation may be achieved in a consultation covering multiple topics.

## Method

### Study setting

We present a qualitative observational single case study intended to explore the subject matter of agenda navigation. By choosing a single case study, we could maintain the overview needed for appropriate adaptation of methodology from linguistics for analysis of a medical consultation. We have conducted a secondary linguistic microanalysis of selected elements from an empirical corpus of 27 video recordings of general practice consultations in Denmark. Recordings were made between 2011 and 2015. Data from 12 GPs (seven women and five men) were initially collected for studies about consultations with patients with multimorbidity and the preventive consultations that are part of how chronic illness is managed in Denmark [20,21]. Most of the consultations in the corpus were annual check-ups for patients with chronic conditions, such as diabetes and cardiovascular diseases, and followed specific national guidelines. The patients were 14 women and 13 men aged 32–82. The consultations were video recorded and lasted from eight to 31 min. In some consultations, only one (health) problem was discussed. This prioritization was either made clear from the beginning, or the GP did not invite the patient’s agenda. In others, the patient talked for a while, and then the GP asked the patient to focus on one specific issue. In yet other consultations, the GP asked if the patient wanted to discuss more issues after plans and agreements had been made about the first subject.

### The case

Variations of agenda mapping and navigation were observed in our corpus of data. Among consultations covering more than one health problem, we chose the most exemplary case, following Gobat et al. [7]. The consultation had the following features: (1) a list of talk topics that were identified at the beginning; (2) an agreement on the focus of what to talk about during the session; (3) the GP raised topics he considered important; (4) the patient raised topics she considered important; (5) the patient was involved and engaged in the conversation, and (6) the conversation was collaborative. Agenda mapping was conducted by the GP from the very start of the consultation when he stated that the main agenda was diabetes control. He also conducted mapping by inviting the patient to talk on topics, asking if she had something else on her mind. This consultation offered variations in agenda navigation strategies in a format conducive to linguistic microanalysis. The selected consultation was a planned annual follow-up of type 2 diabetes lasting 17 min and 45 s. The patient was a 43-year-old woman and the GP was a 63-year-old man. They had an established doctor-patient relationship and both spoke in Danish.

### Theory and analysis

John Langshaw Austin (1911–1960), a British philosopher of language, developed a theory about *speech acts* [22]. According to Austin, *performative speech acts* are statements that constitute or are part of an action: you make a bet by saying ‘I bet’; you promise something by saying ‘I promise’. Still, performatives are often *implicit*, without the act specifically named as part of the utterance. Every performative could in principle be transformed into an *explicit* performative, but one usually does not name the act of, for example, insulting someone when delivering the insult. By saying, ‘You really are an exceptionally resourceful patient’, a doctor performs an act of support and recognition of the patient. Austin calls the act in saying something an *illocutionary act*. Among the examples of typical illocutionary acts, he mentions giving estimates or appraisals and making announcements about intentions. Accepting and informing are other prominent examples. Pragmatic linguistics and Austin’s speech acts have previously been used by Nessa to microanalyze complex consultations in general practice [23], demonstrating the strength of this framework for analyzing medical talk as interaction [24]. Nessa emphasizes that ‘indirect speech acts’ are common in medical discourse [23]. In indirect speech acts people express what they mean in an indirect way, only understandable within the specific context.

To analyze agenda navigation in consultation with multiple health problems we developed a unique analytic framework for microanalysis of the selected case applying theoretical perspectives and concepts from discourse analysis [25] and pragmatic linguistics [22]. The concept of agenda mapping and navigation by Gobat et al. [7] is relying on speech acts [22], even though this is not discussed in detail. We have identified and microanalyzed speech acts indicating a transition between episodes that address different *topics* [19] discussed during the consultation. An *episode* was defined as a brief unit of action [26].

First, we established a speech act-based synopsis (Box 1), using Nessa’s method for transcription to establish an overview of what was going on in this particular consultation [27]. Then we organized the material for analysis by identifying topics in the consultation [19]. According to Ainsworth-Vaughn, new topics lack references to what was previously discussed and are discussed without acknowledgment of previous discourse. In the selected consultation we identified 10 episodes addressing specific topics, most of them related to different particular aspects of diabetes (Table 1). We focused on the use of *performative speech acts* by the GP when navigating the agenda by opening and closing these 10 episodes. Interpretations were negotiated and validated among the three authors.

**Table:** Synopsis presenting overview of the consultation [27]

D welcomes and announces **annual diabetes checkup**, adds ‘Have you thought of something else?’
P requests her **blood test results**, confirming that there is more to be discussed. D accepts by specifying and praising the results, comparing with previous tests P expresses relief ‘That was wonderful’. D appraises the heart examination, no problems, inviting ‘What was on your mind?’
P suggests cutting down a **medication** which the pharmacy cannot procure. D clarifies alternatives, confirming that there is a storage problem. D accepts her suggestion, presents future plans, reevaluation in 3 months, invites for more.
P complains of a **wound** coming and going. D encourages her to show it, recommends ointment: ‘Shouldn’t we try it?’ P hesitates, since it comes back, but accepts the doctor’s suggestion to await the effect.
P announces her **weight** to be checked, adding: ‘I think I have lost weight’. D confirms and appraises weight reduction: ‘That is fine’, asking patient why so.
P presents **diarrhea**, hinting: ‘I really hope there is nothing wrong’. D refers to tests some months ago, repeating positive comments. P agrees, but objects that something might be wrong. D requests information about the symptoms. P describes changes in bowel habits last half-year. D considers bowel investigation if it continues. P presents fear: ‘People say it is really painful’. D explains the examination, toning down discomfort, summarizes changes with no obvious reason, trying to convince the patient about the referral. P accepts referral reluctantly, arguing: ‘It would be unpleasant if there is actually something wrong’. D justifies his recommendation, asking: ‘Shouldn’t we do it?’. P accepts.
D informs that he returns to the annual checkup program, announcing and conducting an **examination of the patient’s feet**, commenting on an old tattoo, approving patient’s information about pedicure.
D announces and conducts **blood pressure** measurement, informing about a satisfactory result.
D requests information from the patient about when she last saw an **ophthalmologist**. P believes it was two years ago D instructs about annual appointments, making an agreement about: ‘You know why, don’t you?’
D requests information about **exercise** from the patient. P responds: ‘I have begun walking my dog again’, 1 h three times a week. D checks the intervals and efforts, praising her: ‘It is good that you have started’. P confirms that she likes this habit, ‘getting crazy just staying home’.
D comments that walking is good for her diabetes, adding: ‘What about **smoking**?’ P confirms that she still smokes 15–20 cigarettes a day ‘… after I started being home’. D asks whether she wants help or if she plans to change. P explains that her intentions are changing, but that it is difficult. D confirms he understands ‘there is something cozy about it’, encouraging her for a new effort to quit, leaving it up to the patient what would happen – ‘… maybe you succeed’.
D starts overall closure announcing and negotiating agreements about future aims for weight and medication until next visit, offering prescriptions and referrals. Saying goodbye. P saying goodbye.

**Table 1.** Outline of episodes concerning specific topics.

Topic no.	Time	Line no.	Introduced by doctor	Introduced by patient
	00:00	000	Opening	
1	00:15	011		Blood tests, results
2	01:47	036		Medication, diabetes
3	03:31	065		Wound after boil
4	04:35	087		Weight
5	05:36	102		Diarrhea
6	10:48	204	Feet examination	
7	11:39	222	Blood pressure	
8	12:43	228	Ophthalmologist	
9	13:24	243	Exercise	
10	14:12	259	Smoking	
	15:41	282	Conclusion	
	17:45	325	End of consultation	

### Research ethics

Informed consent was obtained from all patients. The anonymity of the participant was ensured by omitting identifying information. The Faculty of Health and Medical Sciences, University of Copenhagen, approved the applications of management of personal information in this project submitted 22 January 2019 (ref #514-0285/19-3000 and # 514-0286/19-3000).

## Results

Our microanalysis revealed various aspects of agenda navigation accomplished by the GP using speech acts. We identified opening and closing episodes to be important communicative strategies for a consultation like this one that covered several issues. The GP opened episodes using speech acts to accept the patient’s request to discuss topics, and these occurred mainly at the beginning of the consultation. Speech acts to inform or to request information from the patient dominated later in the consultation. The GP closed all episodes, using speech acts to instruct or appraise the patient, or to make agreements or plans.

### Opening episodes by accepting, informing, and requesting

The GP opened episodes using different speech acts, such as accepting the patient’s request to discuss or to check something, informing the patient that some topics, such as blood pressure measurement are part of diabetes control, and requesting information from the patient.

Opening a new episode by more or less indirectly accepting the patient’s request was seen in several instances. In one example, it was the recognition of the need to discuss something, as when the patient stated that she would like to know the results of tests:

*P: …but I’d rather know the results first.*

*D: Yes.*

*P: I do actually have something that I’d like to discuss.*

*D: Yes.*

*P: But, well, I don’t know what the results are.*

*D: They are fine.*

*P: I was hoping so.*

*D: Well, they are. You had these blood tests…*

(Episode 1, Blood tests, results)

In the last sentence, the GP has accepted the patient’s wish to talk about blood tests and opens a new episode on this topic. Another episode was opened by encouraging the patient to present a wound which she was concerned about, ‘*Where was it now?*’ Speech acts accepting the patient’s request to talk about a specific topic and to do clinical examinations were used by the GP to open new episodes early in the consultation.

Later in the consultation, the opening of episodes was dominated by speech acts from the GP informing the patient that some topics were part of diabetes control, or calling for further information from the patient. For example, a new episode was opened when the GP notified the patient that he wanted to examine her feet as part of diabetes control at the annual check-up:

*D: Well, I’m actually expected to examine your feet.*

*P: Yes.*

*D: It’s part of the set up.*

(Episode 6, Feet examination)

The GP’s requests for information from the patient as a way to open an episode were seen when he introduced topics about exercise and diabetes and topics for follow-up by other professionals.

A request for information was also observed when the GP opened a topic that had not been mapped from the beginning of the consultation. The patient had tried to lose weight before, but now she had unexpectedly lost six kilos. Ambiguity regarding the significance of this weight loss emerged since she also had persistent diarrhea:

*D: Well, that’s a good development.*

*P: It’s nice that I lose weight. I really hope there is nothing wrong.*

*D: We had your stools examined for blood and that was normal though. But you still have diarrhea?*

(Episode 5, Diarrhea)

The patient delivered an emotional cue, by expressing worry that something might be wrong with her bowels to cause weight loss. By requesting information from the patient about diarrhea, the GP performed the illocutionary act of opening a new episode that was not agreed upon initially. This topic, diverging from the different diabetes issues, was allocated a substantial amount of time in the consultation (5 min 12 s). Still, the GP’s navigation did not displace the remaining issues in the annual check-up.

### Closing episodes by instructing or appraising the patient, or making agreements or plans

In the selected consultation, the GP closed every topic before a new episode was opened using an array of speech acts for this purpose. He closed by concluding that the patient’s test results and activities were satisfactory, by making plans for treatment or further investigation, by making agreements with the patient, by giving instructions about future follow-up, or by passing on responsibility for further action to the patient.

The speech act in this dialogue demonstrates the closure of an episode about test results:

*D: And then you had an electrocardiogram made, you know an examination of the heart, which was also normal.*

*P: Yes.*

*D: So no problems in that field, right.*

(Episode 1, Blood tests, results)

‘No problem’ answers around tests like this one are interpreted as speech acts of appraisal, here functioning as closure. Similar appraising performatives were used for processes that seemed to be going in the right direction, such as when initially assessing the patient’s weight loss as positive.

Presenting future plans was used to close another episode concerning adjustments to the patient’s medication:

*D: We can try. There are no problems in that. And then make an assessment in three months and have a look at the results. And then we could consider other medications. If any of the new medications are better.*

*P: Yes.*

(Episode 2, Medication, diabetes)

Similarly, the GP’s plans for treatment closed an episode concerning the residual ulcer after treatment of a boil.

The closing of a topic by giving the patient instructions and of making an agreement was done, for example, after discussion of the patient’s visits to an ophthalmologist as part of diabetes follow-up. Comparable agreements were made for many topics and were confirmed at the end of the consultation.

The speech act of leaving it up to the patient about what would happen next was used to close an episode concerning smoking, which the patient was not yet ready to quit:

*D: So you do fight a little.*

*P: I quit for one year and for one and a half. And then nevertheless you start again. Well, it’s stupid.*

*D: That’s how it is. Then you’ll just have to wait and see if suddenly you feel like quitting again.*

(Episode 10, Smoking)

## Discussion

Microanalysis revealed the impact of opening and closing episodes as communicative strategies suitable for the GP in patient-centered communication about several topics. Episodes were opened by speech acts accepting, informing, and requesting; while instructing or appraising the patient, or making agreements or plans were used for closing episodes. Below, we discuss the impact of these findings.

### What is known from before, what does this study add?

Multimorbidity challenges ideas of the ‘normal’ consultation covering only one topic [28], highlighting the insufficiency of prevailing conceptual frameworks that simplify the tasks of the doctor. Our analysis demonstrates how a GP may use speech acts to organize a consultation addressing more than one health problem. Traditional consultation models present phases in a process to address a single health problem [29,30] and then describe further issues raised in consultation as ‘additional concerns’ [31]. In clinical reality, the discussion of more than one topic is unavoidable as patients’ symptoms may relate to more than one illness, illnesses might affect each other or treatments could interfere.

The concepts of *concordant* and *discordant* illnesses designate combinations that are treated together and share some risk factors and, on the other hand, illnesses that are unrelated in pathogenesis or management [32]. Health problems in the consultation we analyzed are both concordant and discordant in these terms. Weight loss surfaces as a potentially concordant issue since it is desired from a diabetes perspective, but in this case, the talk indicates that it may represent a discordant health problem. It may be a symptom of bowel disease and not a success of diabetes self-care and is given further attention in its own right. To arrive at this knowledge, however, the GP needed to include both possibilities in the consultation.

Furthermore, our interpretations suggest that the continuous closing of episodes by the GP contributes substantially to the orderliness and feasibility observed in this consultation. Closing has been studied in terms of the end of a medical visit [4], where it is usually tied to a ‘chief concern’ [33,34]. Our analysis demonstrates the closing of several episodes in the same consultation, and that some were closed by means similar to those used in closing a consultation, such as making a plan for future care. In previous concepts of agenda-setting, prioritizing what to discuss in a consultation is presented as a core issue, but to our knowledge, closing has not been specifically considered in this context.

We suggest that highlighting the opening and the closing of several different topics should be seen as an integrated part of agenda-setting in consultations with multiple issues. Interventions targeted at patients with multimorbidity often offer more time and explicitly encourage the inclusion of all the patient’s health problems, but a consultation strategy is usually not specified (e.g. [35,36]). Our study adds the skills of opening and closing individual topics by speech acts to a consultation process suitable for the handling of several health problems.

Botelho has previously drawn attention to the idea that agenda setting happens throughout a consultation, not just as an upfront activity [37]. In this article, we take this point even further by emphasizing and extending the impact of agenda navigation, and proposing that such navigation is a core competence for the GP. Upfront agenda setting assumes that all topics can be revealed and prioritized from the beginning. As demonstrated in our case some issues may, however, emerge during the consultation, as the discussion of one health problem leads to other health problems. It is difficult for both the GP and the patient to foresee which issues will be touched upon, as symptoms may stem from several health problems, and treatments may be interacting.

Our case illustrates a strategy, possibly exercised unconsciously or habitually by the doctor, that allows the consultation to cover several issues, some of which were allocated considerable time and attention, even the potentially discordant issues. We argue that this requires experience and skill, the latter being made more accessible for analysis and teaching using description and reflection. Descriptions and analysis of practical knowledge among GPs around consultations that cover more than one health problem should inform the teaching of medical students and physicians in specialty training as part of a formal curriculum. This would help to integrate appropriate knowledge developed specifically for this context in medical education and make it accessible for professional discussion.

### Strengths and limitations

We chose to conduct a single case observational study [38] to perform a thorough microanalysis of linguistic interaction [22,23]. Instead of doing a thematic analysis across several of the available consultations, this design allowed us to present an overview of a complete consultation where the GP accomplished agenda navigation across several topics. We found the methodological tools adequate and easy to adapt to this format. By selecting a case from a larger corpus, we were able to identify, assess and present several hallmarks of similarity, as well as of contrast, in the specific case in the context of the complete corpus. This was not a comparative pursuit but a systematic approach to differentiate the exemplary features of agenda navigation observed in the case.

Regarding demography, this case was not exceptional in terms of patient-doctor dyads comparable on gender, age, background, and culture in the corpus. The type and content of medical issues discussed in the consultation were also unexceptional. The discourse in the case, compared to several of the other cases in the corpus, was distinguished by proceeding in an orderly and well-organized manner, frequently involving the patient and a consultation ambience with a low level of conflict. As such, we recognize this specific case is not necessarily representative of consultations in general practice, while it still holds strong internal validity for the issues we intended to explore [39]. The external validity of what is going on is probably limited to consultations comparable to this one, which was exceptionally and exemplary well-organized without conflicts. Nevertheless, in the sense of what can be learned and transferred from our analysis to other consultation types, we appraise the external validity of our study as strong. The consultation lasted 17 min 45 s and covered ten topics. Many consultations in general practice are shorter and cover fewer topics, and some consultations are even more complex. Principles for navigating the agenda are, however, transferable to other types of consultation.

Concepts and perspectives from speech act theory [22] and topic division [19] provided access to the identification of implicit and explicit markers of agenda navigation [7], some of them rather subtle. Our interpretation of which acts were performed was probably more supported than disturbed by our own experiences from clinical practice, perhaps especially what we were able to recognize as indirect speech acts. We decided not to evaluate systematically the communicative quality of the dialogue, to better grasp the descriptive patterns of navigating. Another choice was to emphasize the GP’s navigation moves, rather than assessing the interaction as such. Although the GP was mostly in charge of the agenda we agreed, upon an overall review of this consultation, that the atmosphere was mutual and collaborative.

## Conclusion and practice implications

Presenting this study, we highlight the fact that consultations in general practice often cover multiple issues. Consistent with the core values of general practice, this is not something to prevent or avoid. The GP must on the contrary be prepared to encounter a diversity of problems, although the complete list cannot always be solved there and then. This is a regular challenge where skillful agenda navigation may become an important tool for patient-centered management and quality of care.

Our methodology offers concepts, perspectives, and examples for how relevant speech acts can be recognized, exercised, and shared, demonstrating how awareness and willingness to handle several topics are manageable using proficient talk. By considering the consultation as a series of episodes where individual topic changes are distinctly indicated by opening and closure, GPs may individually elaborate their speech acts and contribute to more space for interaction with patients.

Our findings should be refined and improved in further research, to develop teaching tools for further differentiation and implementation. They may, however, prove to be useful in an everyday practice setting already at this point.
